# Experiments with Seasonal Forecasts of ocean conditions for the Northern region of the California Current upwelling system

**DOI:** 10.1038/srep27203

**Published:** 2016-06-07

**Authors:** Samantha A. Siedlecki, Isaac C. Kaplan, Albert J. Hermann, Thanh Tam Nguyen, Nicholas A. Bond, Jan A. Newton, Gregory D. Williams, William T. Peterson, Simone R. Alin, Richard A. Feely

**Affiliations:** 1 Joint Institute for the Study of the Atmosphere and Ocean, University of Washington, Box 355672, 3737 Brooklyn Ave NE, Seattle WA 98195 USA; 2 Conservation Biology Division, Northwest Fisheries Science Center, National Marine Fisheries Service, National Oceanic and Atmospheric Administration (NOAA), 2725 Montlake Blvd E, Seattle WA 98112 USA; 3 NOAA, Pacific Marine Environmental Laboratory, NOAA, 7600 Sand Point Way NE, Seattle WA 98115 USA; 4 Applied Physics Laboratory, University of Washington, 1013 NE 40th St, Box 355640, Seattle, WA 98105 USA; 5 Pacific States Marine Fisheries Commission, under contract to Northwest Fisheries Science Center, National Marine Fisheries Service, NOAA, 2725 Montlake Blvd E, Seattle WA 98112 USA; 6 Fish Ecology Division, Northwest Fisheries Science Center, Hatfield Marine Science Center, Newport, Oregon 97365 USA

## Abstract

Resource managers at the state, federal, and tribal levels make decisions on a weekly to quarterly basis, and fishers operate on a similar timeframe. To determine the potential of a support tool for these efforts, a seasonal forecast system is experimented with here. JISAO’s Seasonal Coastal Ocean Prediction of the Ecosystem (J-SCOPE) features dynamical downscaling of regional ocean conditions in Washington and Oregon waters using a combination of a high-resolution regional model with biogeochemistry and forecasts from NOAA’s Climate Forecast System (CFS). Model performance and predictability were examined for sea surface temperature (SST), bottom temperature, bottom oxygen, pH, and aragonite saturation state through model hindcasts, reforecast, and forecast comparisons with observations. Results indicate J-SCOPE forecasts have measurable skill on seasonal timescales. Experiments suggest that seasonal forecasting of ocean conditions important for fisheries is possible with the right combination of components. Those components include regional predictability on seasonal timescales of the physical environment from a large-scale model, a high-resolution regional model with biogeochemistry that simulates seasonal conditions in hindcasts, a relationship with local stakeholders, and a real-time observational network. Multiple efforts and approaches in different regions would advance knowledge to provide additional tools to fishers and other stakeholders.

Ecological forecasting is considered a key science capability required to support US coastal ecosystems into the future^1^,^2^. In addition to projections of changes in ecosystems and ecosystem components responding to environmental drivers, ecological forecasts also provide information relevant to how economies and communities may be affected. Ecological forecasts are considered a top priority for aiding ecosystem-based management (EBM), an integrated management approach being implemented by the US National Oceanic and Atmospheric Administration (NOAA) that recognizes the full array of interactions within social-ecological systems^3^. Different types of ecological forecasts help coastal managers and scientists make better decisions based on what may lie ahead at various spatial and temporal scales. Regional associations of the U.S. Integrated Ocean Observing System (IOOS) can offer a critical link between stakeholders (e.g., managers, fishers), NOAA’s EBM efforts, and scientists by disseminating results. To date, most forecasting efforts have been devoted to establishing real-time assessment and short-term forecasts of upper ocean physical properties, and investigating probable future trends on climate time scales^4^,^5^. In contrast, less progress has been made in developing forecasts on the scale of months to a year for ecological variables.

There is an acute need for linked oceanographic and biological resource forecasts on time scales from a few weeks to seasons. Resource managers at the state, federal, and tribal levels must make decisions on a weekly to quarterly basis, for instance to adjust salmon harvest rates or to close areas to crab fishing (http://www.dfw.state.or.us/mrp/salmon/updatesnew.asp). Fishers make decisions on a similar timeframe, for instance deciding what quota or permits to buy or lease, and what port to operate from for a given season^6^. Since marine organisms and ecosystems respond strongly to climate and physical forcing, for instance in the Northern California Current ecosystem off the US West Coast^7^,^8^,^9^,^10^,^11^,^12^, prognostic information could yield substantial benefits for both managers and stakeholders.

Seasonal forecasts of ocean conditions are now possible with output from large-scale seasonal forecast systems. One such system, the Climate Forecast System (CFS), is downscaled through the Regional Ocean Modeling System (ROMS) here. The CFS resolves the global atmosphere at ~200 km resolution and the global ocean at ~50 km resolution^13^,^14^. Monthly and daily averages of relevant atmospheric and oceanic properties are available online (https://www.ncdc.noaa.gov/data-access/model-data/model-datasets/climate-forecast-system-version2-cfsv2), and include both hindcasts from 1979 to present, and forecasts out to nine months into the future. NOAA is currently running the CFS operationally for seasonal weather prediction. ROMS is well suited to resolve small-scale coastal phenomena, and has been successfully used in a wide range of regional studies worldwide^15^. Here, we use ROMS as the link from short-term climate forecasts to ecological processes relevant to fishery managers and stakeholders, and to an Integrated Ecosystem Assessment^16^,^3^. Further, we provide forecasts through an IOOS regional association, the Northwest Association of Networked Ocean Observing Systems (NANOOS), in order to promote and evaluate results to determine the usefulness of the information with Pacific Northwest managers, fishers, and scientists.

Our work mirrors similar seasonal forecasting efforts for Australian marine resources, where a coarser scale (2 degree x 0.5–1.5 degree) predictive ocean-atmosphere model has been developed^17^. There, seasonal forecasts predict distributions of southern bluefin tuna (*Thunnus maccoyi*)^6^,^18^, water temperatures at salmon aquaculture sites^19^, and air temperature and precipitation at prawn farms^20^. One of the key steps in the Australian research has been to evaluate and convey skill and accuracy of the forecast products^21^, which will be our focus below. Another key lesson of these Australian case studies is that a forecast system must focus on specific predictable phenomena that trigger stakeholder decisions. Examples of these phenomena include shifts in the northern extent of tuna habitat as predicted by SST, which shifts the potential hotspots for unwanted bycatch of tuna, or extreme temperatures or precipitation above the upper tercile of historical events, which can be detrimental for aquaculture. In our study, one aim in the management context is for the forecasts to serve as an early warning to Canadian and US managers and fishers of shifts in migration of sardine^22^.

The Pacific Northwest region of the California Current System (CCS) benefits from key components that make forecasting possible, including a skillful high-resolution regional model and predictability in the local oceanography. Compared to other regions in the CCS, this region has a more linear coastline, and stronger seasonal variability in the winds^23^,^24^,^25^. These characteristics shape the predictable seasonal response of the ocean environment. In addition, the lack of relatively unpredictable mesoscale features such as filaments and eddies in the region make it particularly well suited for forecasting. Finally, the northern portion of the CCS also benefits from substantial predictability on seasonal time horizons, as forecast by CFS^26^, but the mechanisms contributing to that predictability require further analysis.

The linked forecasting system discussed here (J-SCOPE, JISAO Seasonal Coastal Ocean Prediction of the Ecosystem) looks to provide seasonal forecasts of ocean conditions that are testable and relevant to management decisions for fisheries, protected species, and ecosystem health. To that end, here we force four different realizations of regional ocean-ecosystem forecasts (2009, 2013 (2), 2014) with CFS to begin exploring how CFS forecast skill translates to high-resolution prediction of the seasonal evolution of physical and biogeochemical anomalies. As described in more detail below, J-SCOPE has reasonable skill to forecast ocean conditions, for instance predicting the development of hypoxic conditions on the Washington shelf in the summer of 2013.

Below we discuss performance and predictability skill of the system. This skill is assessed through the comparison of forecasted anomalies with hindcast anomalies over 2009, 2013, and 2014 in addition to comparisons with *in situ* 2013 observations in the Supplemental Information (SI). We highlight methods for communication of results and potential applications of forecasted ocean conditions to fisheries.

## Methods

J-SCOPE forecasts use CFS output to force a high-resolution ROMS model that includes biogeochemistry. The system predicts the timing of the spring transition, the cumulative upwelling index, SST, chlorophyll stock, dissolved oxygen, pH, aragonite saturation state, and Pacific sardine habitat. Terminology used henceforth includes forecasts (forcing without data assimilation), reforecasts (similar to a forecast, but performed on a past year), hindcasts (forcing with data assimilation), climatology (time-averaged field detailing the seasonal cycle), and anomalies (differences from the climatology).

### Climate Forecast System

The CFS is a global, coupled atmosphere-ocean-land model, which uses a 3DVAR technique to assimilate both *in situ* and satellite-based ocean and atmospheric data^13^,^14^. The CFS resolves the global atmosphere at ~200 km resolution and the global ocean at 25–50 km resolution. CFS output is available online (http://cfs.ncep.noaa.gov/), with forecasts out to nine months, hindcasts from 1979 to 2009 (the CFS-R reanalysis), analyses of 2010–present (the operational CFSv2), and reforecasts of 1979–2009 (CFS-RR). The horizontal resolution of the CFS-R atmospheric reanalysis (38 km) is much finer than that of the CFS (200 km).

CFS outputs alone are potentially useful to fishery managers and stakeholders. These include metrics of ENSO (El Niño-Southern Oscillation^27^), the PDO (Pacific Decadal Oscillation^28^), and regional upwelling indices. Regional upwelling indices are forecasted using the atmospheric wind forecast for the region from CFS and the local upwelling relationship^29^.

### ROMS

**Figure 1:**
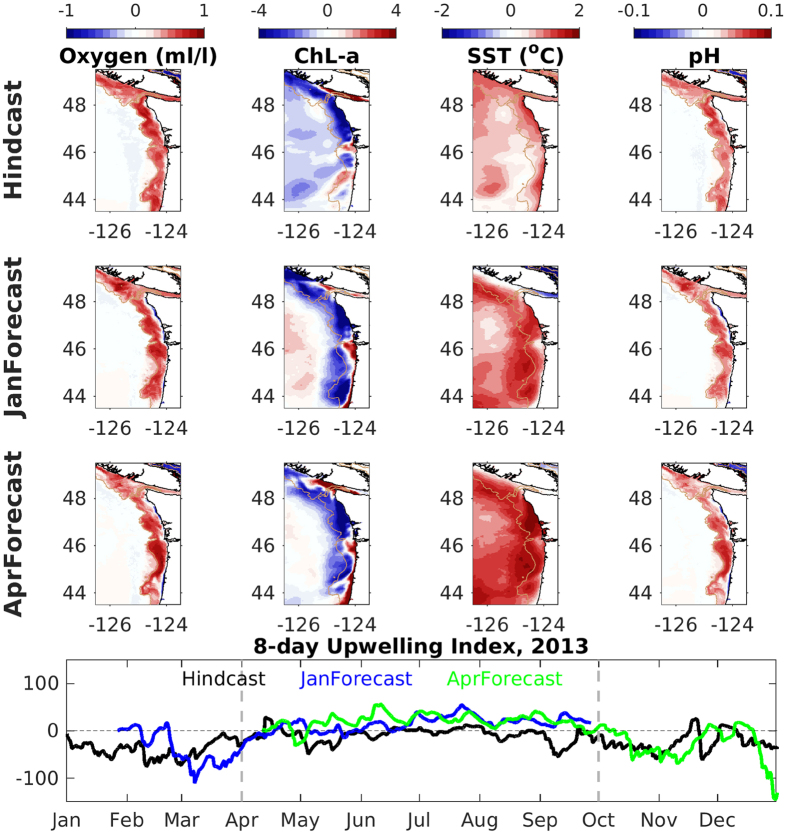
Forecast and hindcast simulated anomalies of the J-SCOPE forecast system averaged over the upwelling season (April–September) from 2013. The hindcast anomaly is on top, the January initialized forecast anomaly in the middle, and the April initialized forecast anomaly on the bottom. Each column displays a different model field. The first column shows bottom oxygen (ml/l), the second, surface chlorophyll, the third is SST, and the last column is bottom pH. Below the maps, a time series of the 8-day upwelling index (calculated following methods from Austin and Barth 2002) is plotted from the hindcast (black), the January initialized forecast (blue) and the April initialized forecast (green). The vertical dotted lines on the time series bracket the upwelling season over which the maps are averaged. Figure generated using Matlab version 2015b (http://www.mathworks.com/products/new_products/latest_features.html) and Adobe Illustrator CS5 (http://www.adobe.com/products/illustrator.html).

The Regional Ocean Modeling System (ROMS; Rutgers version 3) is configured for the Washington, Oregon, and British Columbia coasts, using the Cascadia domain^30^. The domain (Fig. 1) extends from 43°N to 50°N with a horizontal resolution of 1.5 km, and 40 vertical levels. In the hindcast simulations, 16 rivers are based on observed streamflows^30^, but in the reforecast and forecast runs, the rivers are forced using a climatology of local river discharge data over eight years (2000–2007). Tides are included. The biogeochemistry model is described in previous studies^24^,^31^.

Water entering the domain at the southern and western boundaries is specified by CFS. Biogeochemical boundary conditions were implemented as described elsewhere^24^,^31^, using local empirical relationships with salinity. We applied these relationships to generate the initial and boundary conditions as predicted by CFS salinity fields.

### Experiments

Regional hindcast simulations were performed spanning 2009–2014, reforecast mode for 2009, two forecasts for 2013, and one for 2014. Two January initialized forecasts and two April initialized forecasts were test from different years. Forecasts were nine-month projections, requiring six days of computer time on 96 cores. In hindcast mode, the forcing was derived from the CFS-R reanalysis (for 1979–2009), which includes data assimilation, and the CFSv2 analyses (for 2010–present). An important implication of the resolution and the data assimilation is that the simulations using the reanalysis product will be superior to those based on the coarser fields available for forecast forcing from the CFS model.

In this study we applied interpolated CFS results directly to ROMS as surface forcing, boundary conditions, and initial conditions, without bias correction. As a result, our regional model will inherit some of the biases of the large-scale CFS simulations. It is recognized that various methods exist for bias correction, and future versions of J-SCOPE may employ such methods.

January and April were chosen as initialization months in order to test whether 2–4 month predictions could forecast critical periods for fish stocks. Specifically, January forecasts of the spring transition have the potential to predict the onset of upwelling and recruitment success of salmon and many ‘spring spawning’ rockfish, while April forecasts have the potential to forecast the extent of northern migrations of major commercial species such as Pacific whiting (*Merluccius productus*) and Pacific sardines (*Sardinops sagax*).

Based on the ROMS output of oxygen and temperature, we applied regional proxy relationships^32^ that predict aragonite saturation state. Results were compared with observations made on coastal cruises performed in the summer of 2013^33^. From the modeled fields we calculated the percent of the upper 100 meters of the water column that was undersaturated with respect to aragonite. The upper 100 meters was specified because it is the approximate vertical range of the pteropod, a calcifying zooplankton affected by ocean acidification in this region^34^.

### Skill Assessment and Predictability of CFS in the J-SCOPE domain

As a prelude to assessments of J-SCOPE forecast skill, we examined six-month forecast skill of CFS forcing within the J-SCOPE domain. For this comparison a subset of the CFS reforecast data was compared with corresponding values from the CFS Reanalysis (CFS-R). For the reforecasts, we utilized four realizations of the forecast system from the 15^th^ of January of each year, spanning the period 1997–2009 (the period during which ARGO data were assimilated), and examined results of the six-month forecasts of July as compared with the corresponding CFS-R values (52 realizations). We considered four monthly mean quantities in this comparison: SST, density at 40 meters depth as a metric for upwelling, shortwave radiation, and alongshore surface winds. We compared persistence skill with model forecast skill, in order to assess whether the CFS model outperforms one that assumes an average seasonal cycle with temporal autocorrelation in the anomalies. For SST and density at 40-meters, we also correlated reanalysis tendency (i.e. change) from January to July with predicted tendency over that period. The 40-meter density metric was chosen for three reasons: 1) subsurface values are a better metric of upwelling than near-surface values, as vertical velocities are greater at depth; 2) 40 m is largely insulated from diabatic seasonal change; 3) CFS-R has a sea surface salinity bias due to an excessive relaxation to climatology^35^.

In each case we compared average forecast July values with average July reanalysis values (mean bias), correlated forecast July values to July reanalysis values (potential forecast skill), and correlated July reanalysis values to January reanalysis values (persistence skill). For SST and density at 40 meters, we also correlated reanalyzed change from January to July with predicted change over that period.

### Skill Assessment and Predictability of J-SCOPE

Hindcast skill and predictive skill assessment of the J-SCOPE model were based on comparisons to observations from *in situ* cruise data spanning 2009–2014, and at two moorings from the Olympic Coast National Marine Sanctuary (OCNMS) in 42 and 15 meters of water. In 2013 an additional mooring (NANOOS Cha’ba) in 90 meters of water off the northern tip of Washington’s outer shelf was available and we included it in comparisons summarized in the SI (Figure S4). Both the model and the observations were smoothed with a moving average filter of 30 days to focus on seasonal timescales.

To quantify model performance, the model climatology was compared to the co-located observational climatology. Time-series comparisons are presented at the locations of two OCNMS moorings on the Washington shelf (CE042, CE015) as well as three profiles along the Newport line from the inner, mid, and outer shelf of Oregon and temperature time series from NH10^36^,^37^. The moorings were deployed during a portion of the year, and so the comparisons among climatologies are limited to the period the observations were available (May through October). The OCNMS mooring records were averaged over 2004–2014 for temperature and 2006–2014 for oxygen. The Newport line observations (2009–2014) are made bi-weekly year-round, and here we present CTD measurements of temperature and oxygen at three locations on the Oregon shelf: NH03, NH10, and NH25. In addition, we compare with SST (2 m) and BT (70 m) temperatures from NH10 (2009–2014). The model fields were extracted from the same location as the observations from each year, and then those time series were averaged together to make a model climatology for that location.

We use the hindcast simulation of 2009–2014 as a proxy for a long-term climatology. We tested this assumption by comparing the physical forcing from the long-term climatology from CFS-R to the six years used in this study. The shorter climatology is representative of the longer-term climatology for SST and alongshore winds in the model domain (Figures S1 and S2).

To quantify predictability of the forecasts, the forecasted anomalies were compared to the hindcasted anomalies from 2013 and 2009 at the same OCNMS and Newport locations. The residual time series convey qualitative information about how model skill declines with time. Anomalies were generated by subtracting the model climatology results from the forecasts and hindcast over the same time period. We calculated three skill metrics when comparing the observed and simulated climatologies and the forecasted and hindcasted anomalies (see SI for equations): the normalized unbiased Root Mean Squared Deviation (RMSD^38^), the correlation coefficient (R-value), and the normalized Bias. The RMSD is influenced by both the phasing of the series and how well the hindcast (forecast) variability compares with the observed (hindcast) variability. RMSD is normalized by the standard deviation of the reference field (either the observations or the climatology) (Eq. 2, SI). As defined here, the normalized total RMSD value also informs whether the model’s standard deviation is larger (RMSD > 0) or smaller (RMSD < 0) than the standard deviation of the reference field RMSD. As the R-values approach 1, the phasing between the two temporal signals are in agreement; note however that this metric alone does not indicate the correspondence between the magnitudes of the two signals. Finally, we report bias between the model and reference fields. Summary statistics averaged over the upwelling season (April –September) are reported in Table 1 and equations for the skill metrics are available in the SI. Additional information about the data sets used is available in SI Table 1.

## Results

J-SCOPE forecast results are available on NANOOS website bi-annually for stakeholders and the public. Results from the 2013 forecasts and hindcast are shown in Fig. 1 for SST, bottom oxygen, chlorophyll, and pH anomalies over the upwelling season, along with the wind forcing. The forecasted anomalies all indicate the right direction and in some cases, spatial patterns consistent with the hindcast anomaly. A summary of the skill statistics is given in Table 1 and below we discuss the performance and predictability skill of these forecasts. These skill assessments help to convey the level of uncertainty in the forecasts, and how this varies for each ocean variable.

### Hindcast and reforecast skill of CFS, for our region

J-SCOPE forecasts rely on output from CFS for the climate forcing, so it is worthwhile considering the quality of CFS predictions for context. CFS has measurable skill in predicting SST in the Northeast Pacific for lead times of less than six months^26^,^39^, and for the spatial mean SST of the CCS, skill is typically greater than simple persistence when predicting spring temperatures from previous months. Much of this skill resides with the region’s systematic response to both local and remote effects of ENSO associated variability^26^. Prior work^39^ noted that model skill in the CCS rarely exceeded persistence with 90% significance; however, those spatial averages include both the Pacific Northwest (PNW) (where figures^26^ suggest skill greater than persistence) and southern California (where figures^26^ suggest skill less than persistence).

**Figure 2:**
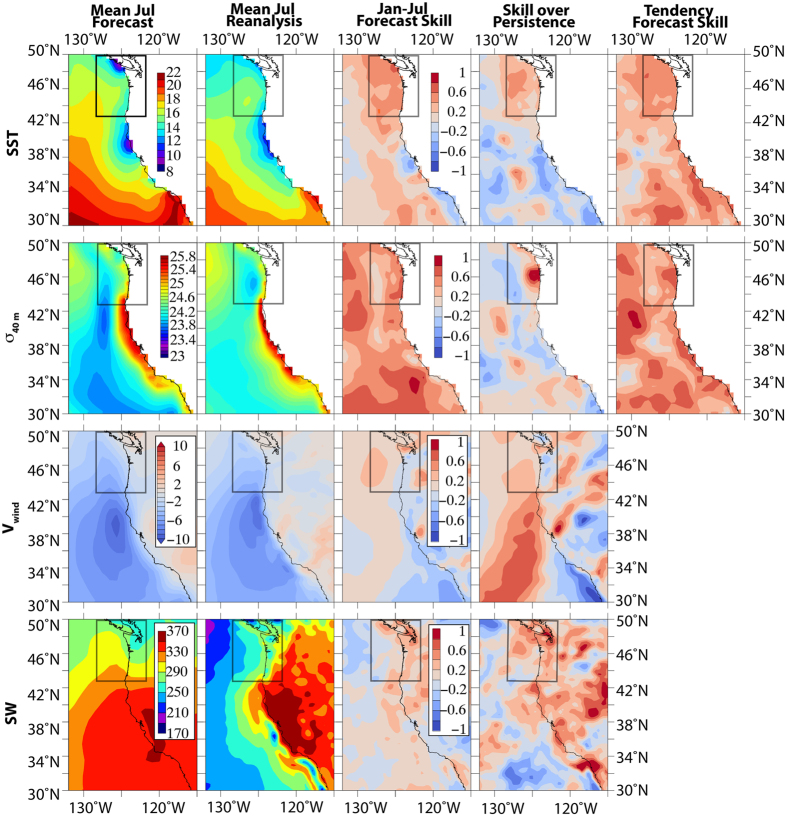
Comparisons for CFS forecasts from 1997 to 2009 and the hindcasts from that same period. Mean fields and skill metrics for sea surface temperature (SST, °C), density at 40 meters (σ _40m_, kg/m^3^), north/south winds at the sea surface (V_wind_, N/m^2^), and downward shortwave radiation at the sea surface (SW, W/m^2^). Boxed region indicates the J-SCOPE domain. Shown from left to right are: 1) mean July forecast (initialized from previous January); 2) mean July reanalysis; 3) “forecast skill” (correlation between July reanalysis and July forecast); 4) “skill over persistence” (forecast skill minus correlation between January reanalysis and July reanalysis); 5) “tendency forecast skill” (correlation of the change from January to July with predicted tendency over that period). The first two columns use the same colorbar (on the first column). The latter three columns use the colorbar in the 3^rd^ column. Figure generated using Ferret version 6.93 (http://www.ferret.noaa.gov/Ferret/) and Adobe Illustrator CS5 (http://www.adobe.com/products/illustrator.html).

Here we take a regional PNW perspective; other studies^26^,^39^,^40^,^41^ provide more quantitative results for the CFS model over an extended period and a broader spatial scale. Consistent with prior results, CFS had substantial skill for the years 1997–2009 and 2013, 2014 when measured against a reanalysis product (CFS-R) (Fig. 2, Table 1). For SST, July forecasts from the previous January have a ~2 degree warm bias near the coast, but achieve useful skill (r~0.4–0.6) in the J-SCOPE domain. Persistence skill is substantially lower in the J-SCOPE domain (r~0.0–0.4, i.e. forecast skill is as much as 0.4 greater than persistence skill in that region; Fig. 2). Forecast skill degrades further south along the California coastline, consistent with the pattern obtained in other studies^26^,^39^ for seasonal predictions of the North Pacific. The PNW may gain some predictability via atmospheric teleconnections with other areas in the Pacific Ocean basin. Our forecast skill values for the Jan-July change in SST (i.e. the 6-month average rate of change of SST over that period) are higher than the forecast skill values for July SST.

For density at 40 meters, the forecast values near the coast are slightly greater than the reanalyzed values (Fig. 2). Forecast skill is appreciable throughout the PNW domain, and extends further south than was the case for SST. Persistence skill is substantially lower (and even negative) around the Columbia River outflow; hence the forecast model adds significant value there relative to simple persistence of observed anomalies from January. Forecast skill for the Jan-July change in density is roughly of the same order as the July forecast skill, achieving values of 0.6–0.8 in the PNW domain. This suggests that the CFS is serving as a useful predictor of seasonally integrated upwelling.

**Figure 3:**
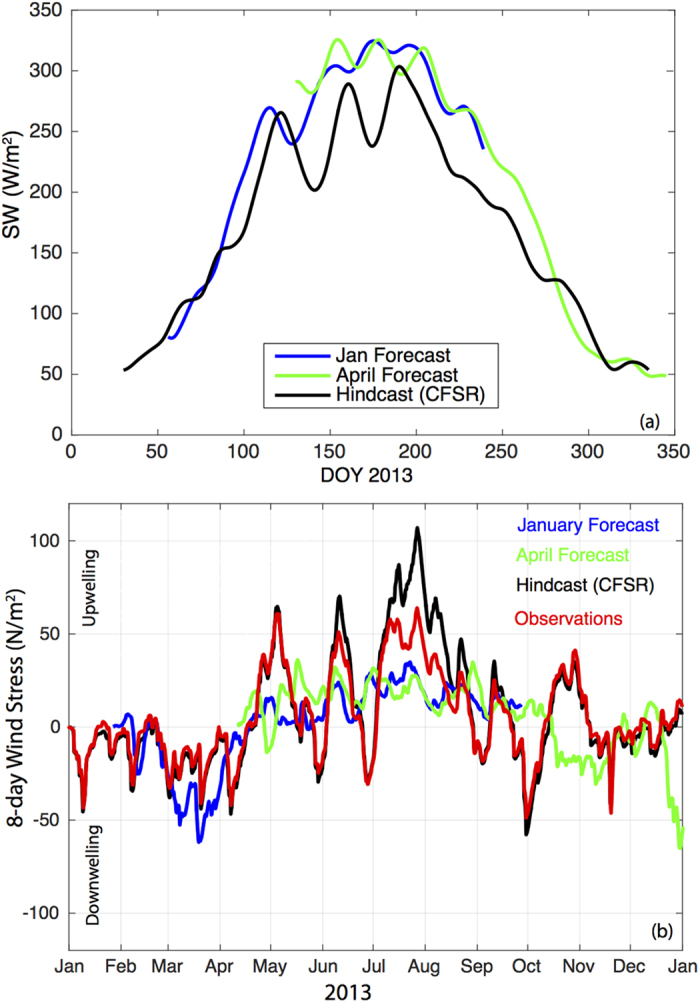
Forecasted and reanalysis product of the (**a**) 15-day averaged shortwave radiation forcing (W/m^2^) for the study region and (**b**) the 8-day wind stress for the J-SCOPE forecast system from CFS for 2013. For the shortwave radiation (**a**), the reanalysis product is in black while the forecasts from January and April are in blue and green, respectively. The CFS model is known to be biased high. For the winds (**b**), the observations are in black, the reanalysis from CFS-R is in blue, the April and January initialized forecasts are in red and green, respectively. The CFS model forecasts miss the duration of the upwelling seasons, as well as the relaxations/reversals in the winds over the upwelling season.

Shortwave radiation, which is the solar energy that warms the ocean surface and drives photosynthesis by phytoplankton, is biased high in CFS, and this bias is inherited by J-SCOPE. For the incident shortwave values, the forecast is biased significantly higher, and a modest correlation exists between reanalysis and predicted July values along the PNW coastline, which exceeds simple persistence (Fig. 2). In 2013, the forecasted shortwave radiation was biased high by about 50 W/m^2^ when averaged over the modeled region (Fig. 3a), and by nearly 100 W/m^2^ in 2009 (not shown). This suggests significant interannual variability in the bias of the CFS, which has not been characterized in our region.

For alongshore winds, the July forecast has a slight increase in skill over anomaly persistence, and is slightly biased towards stronger northerly winds in the PNW, with weak forecast skill at the 6-month lead time (Fig. 2). It remains to be quantified how skillful the CFS forecasts are at capturing the time-integrated wind stress over the upwelling season, as compared to these 6-month forecasts of July winds. Based on observed winds from 2013, the observed summer upwelling season of 2013 began on April 8 (damp.coas.oregonstate.edu)^42^, which is typical, but the season ended 20 days earlier than average. The CFS forecasted winds captured the onset of the upwelling season with the spring transition, and the January forecast was close to the observed cumulative upwelling value for August (not shown). However, the upwelling season was too long and the winds were too strong, without enough relaxations (Fig. 3b).

In summary we find that CFS has measurable skill (relative to a null model of persistence) for two key metrics of ocean conditions that are relevant to our upwelling-driven ecosystem: integrated upwelling and alongshore wind stress. All models are biased and imperfect; however, two key biases are evident in the 2013 CFS forecasts that subsequently influence the J-SCOPE forecasts:

- Shortwave radiation (too much solar radiation)
- Wind Events (upwelling winds persist too late into the fall and too strong with minimal relaxations throughout the upwelling season).

These patterns in shortwave radiation and winds affect predictions, particularly the ability to forecast late summer and fall ocean conditions.

### J-SCOPE skill: performance and predictability

Below we discuss two aspects of model skill: *performance*, based on comparisons between observed and simulated climatologies; and *predictability* based on comparisons of forecasted and reforecasted anomalies to the hindcast anomalies. Performance refers mostly to the ability of the model to predict the seasonal cycle in the region. The results of the predictability metric provide confidence in the model’s ability to generate forecasts on this time period and inform as to what time interval the forecasts have skill. The focus of the figures is on 2013, but the results for forecasts from 2009 and 2014 are included in Table 1 and the discussion of the results below.

#### Model performance

**Figure 4:**
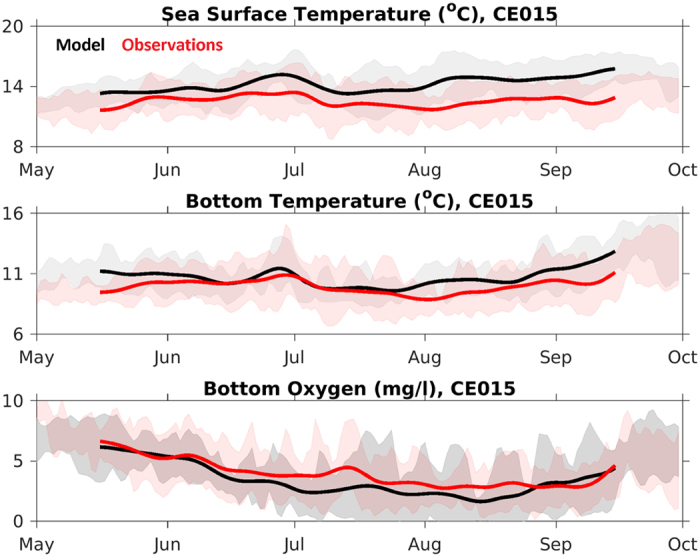
Time series of the observational climatology (red) from the OCNMS mooring location, CE015, off the Washington shelf in 15 m of water. The model climatology (black) is the average of the 2009–2014 hindcasts. The time series have been smoothed with a 30-day filter. The standard deviation around each climatology is shaded in the background. (**a**) SST, (**b**) bottom temperature, and (**c**) bottom oxygen (mg/l). Statistics summarized in Table 1.

**Figure 5:**
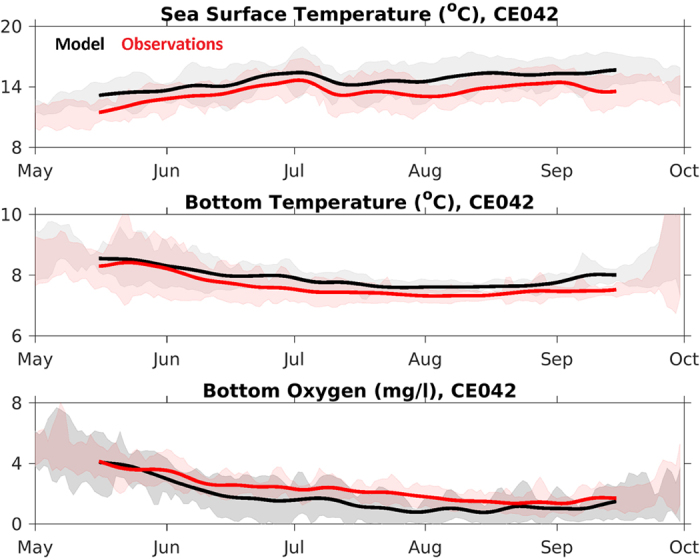
Time series of the climatology from the OCNMS mooring location, CE042, off the Washington shelf in 42 m of water. The climatology uses the 2009–2014 hindcasts averaged together as a reference climatological field. The time series have been smoothed with a 30-day filter. The standard deviation around each climatology is shaded in the background. (a) SST, (b) bottom temperature, and (c) bottom oxygen (mg/l). Statistics summarized in Table 1.

**Figure 6:**
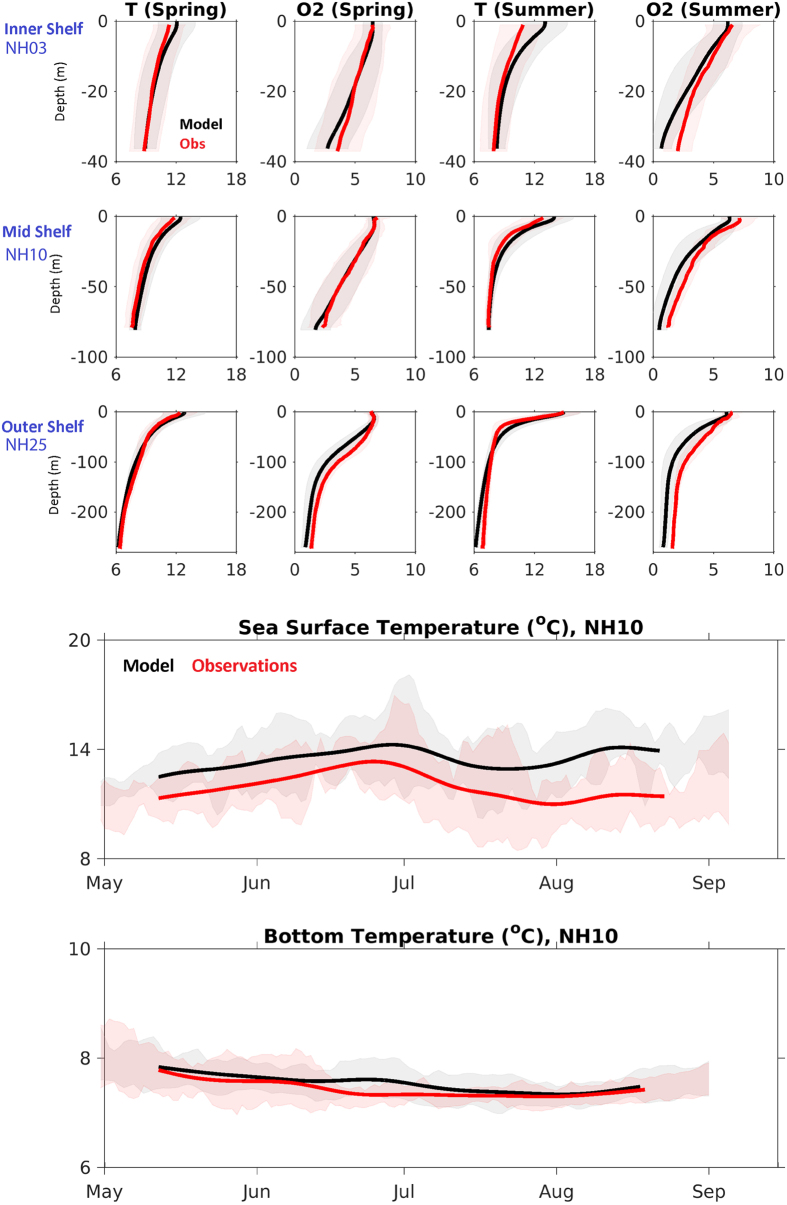
A comparison of model and observational climatologies from three locations (inner, mid, and outer shelf) along the Newport Line on the Oregon shelf. The model climatology (black) is the average of the 2009–2014 hindcasts, at each location and depth. The observational climatology (red) is based on twice-monthly samples. wThe standard deviation around each climatology is shaded in the background. Temperature and oxygen (ml/l) profiles are shown from spring (April-June) and summer (July–September). Fall and winter appear in the Supplemental Information. Statistics summarized in Table 1. In addition, time series from a mooring at the NH10 site (red, 2009–2014) and hindcast climatology (black) are provided in the bottom two panels: SST (2 m, top) and BT (70 m, bottom).

When compared to local observations, J-SCOPE predicts the trends and seasonality of the observed SST, bottom temperature, and bottom oxygen on both the Washington and Oregon shelves: CE015 (Fig. 4), CE042 (Fig. 5), and Newport (Fig. 6). The model captures the seasonal cycle – SST is warmest during the summer, bottom temperature is coldest during the summer upwelling season on the shelf, and bottom oxygen declines over the course of the summer upwelling season. The shaded regions in Figs 4, 5, 6 indicate the interannual variability within the climatology for both the observations and the model, and qualitatively, the model captures that spread as well.

To quantify the agreement between the modeled and observed climatologies, normalized RMSD as well as R-values and bias were computed for the time series and profiles from Figs 4, 5, 6, and the results are compiled in Table 1. Additional winter and fall profiles are available in the Supplementary Information (Figure S3). The model climatology captures the seasonal variability in the observed climatology as indicated by the normalized RMSD values (<1), and has significant R-values for all variables and locations tested except for SST at the shallowest location (CE015, Table 1).

The model simulates bottom temperature more realistically than SST, when compared to observations (Table 1). Though the bottom temperatures are still biased high at all locations, the model captures the variability over the upwelling season, as shown in Figs 4 and 5. Similarly, the simulated bottom oxygen concentrations compare well with observations (Table 1, Figs 4, 5, 6). The modeled bottom oxygen is biased low, but simulates the seasonal decline (Figs 4 and 5) and vertical structure (Fig. 6) at various locations on the Washington and Oregon shelves over the upwelling season.

Skill assessment on individual years, such as 2013, is available in the Supplementary Figures S4 and S5. Results of the skill assessment presented here are similar to the RMSD values from prior hindcast simulations with higher resolution atmospheric forcing^31^. Because the model can simulate the observed seasonal cycle in all three ocean conditions with reasonable skill as a hindcast, the model performs well enough to be tested in forecast mode and its predictability can be tested using similar metrics.

#### Model Predictability

**Figure 7:**
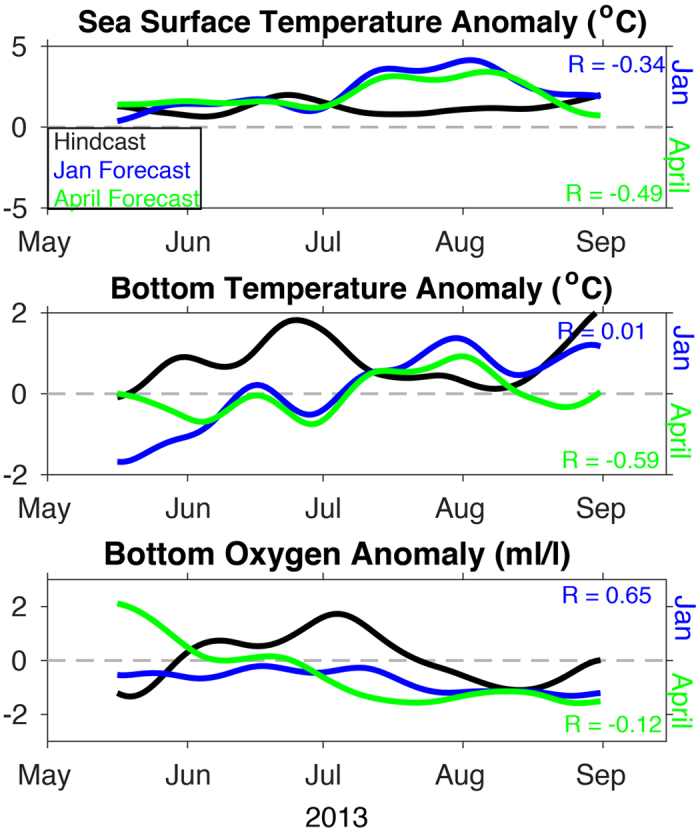
Time series of the anomalies from the OCNMS mooring location, CE015, off the Washington shelf in 15 m of water. The anomalies use the 2009–2014 hindcasts averaged together as a reference climatological field, shown in Fig. 4. All anomalies are for the 2013 forecasts (January initialized is blue, April initialized is green) and hindcast (black). The time series have been smoothed with a 30-day filter. R for each forecasted anomaly is reported on the figure as well as in Table 1. (a) SST, (b) bottom temperature, and (c) bottom oxygen (ml/l).

**Figure 8:**
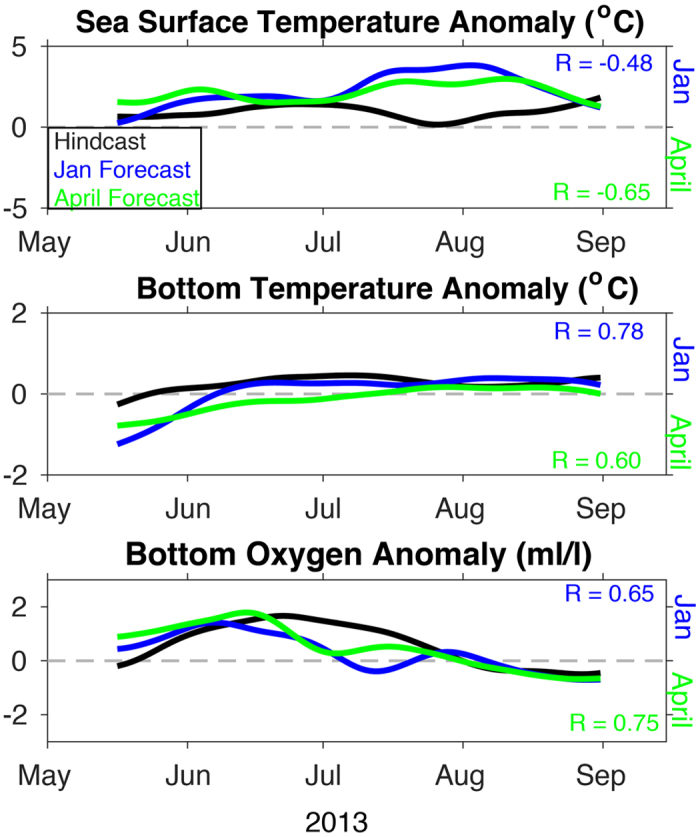
Time series of the anomalies from the OCNMS mooring location, CE042, off the Washington shelf in 42 m of water. The anomalies use the 2009–2014 hindcasts averaged together as a reference climatological field shown in Fig. 5. All anomalies are for the 2013 forecasts and hindcast. The time series have been smoothed with a 30-day filter. R reported on the figure as well as in Table 1. (a) SST, (b) bottom temperature, and (c) bottom oxygen (ml/l).

**Figure 9:**
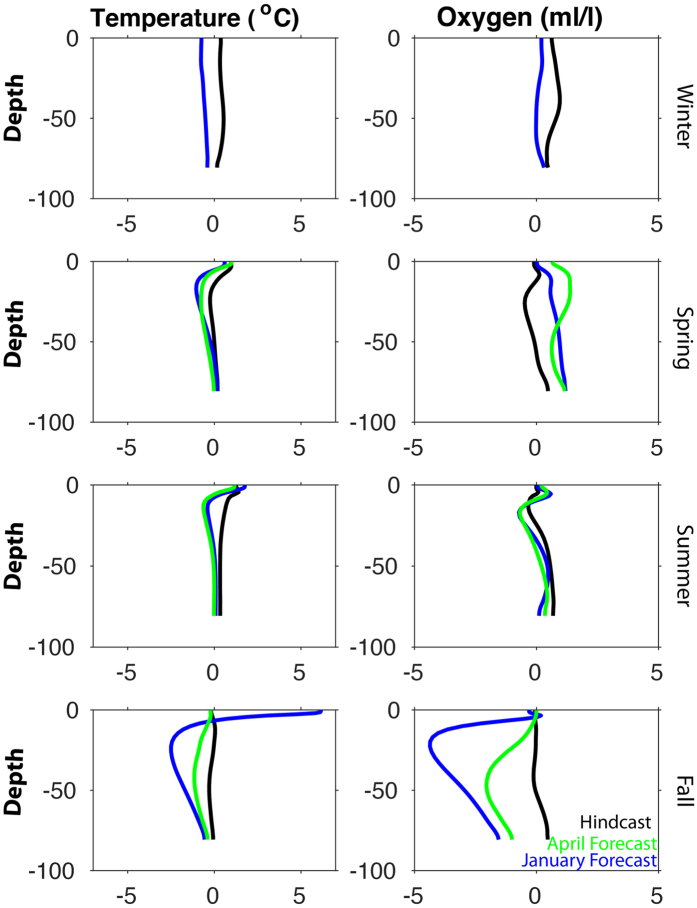
A comparison of forecast versus hindcast anomalies from the NH10 location (mid-shelf) along the Newport Line on the Oregon shelf. All anomalies were calculated as the difference from a climatology based on the average of 2009–2014 hindcasts. January forecast is in blue, the April forecast in green, and the hindcast is in black. Temperature (left) and oxygen (right) profiles are shown from spring (April-June) and summer (July-September), fall (October-December) and winter (Jan-March). Statistics summarized in Table 1.

**Figure 10:**
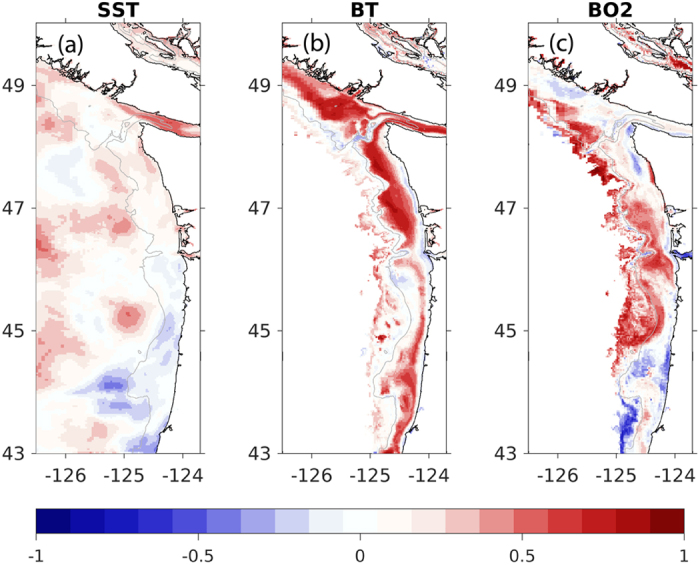
Plot of model performance comparing forecasted anomalies to hindcasted anomalies, for four forecasts and three ocean conditions. Each map shown represents R (as in Table 1), based on six monthly anomaly maps (April, May, June, July, August, September). The comparison demonstrates skill when R > 0.5. Each panel displays a different model field (**a**) SST, (**b**) bottom temperature (°C) (**c**) bottom oxygen (ml/l)). The bottom maps show model fields shallower than 500 meters. All panels highlight the 200-meter isobaths as an indication of the shelf break. Figure generated using Matlab version 2015b (http://www.mathworks.com/products/new_products/latest_features.html) and Adobe Illustrator CS5 (http://www.adobe.com/products/illustrator.html).

The results above focus on performance of the hindcasts in reproducing the seasonal cycle; the general strengths and weaknesses evident in the hindcasts are also apparent in terms of model predictability, assessed by comparison of hindcasts to forecasts of the same period. Qualitatively, the evolution of model forecasts for 2013 matches trends at the two moorings (Figure S4). While these results are promising, it is important to show that J-SCOPE has skill beyond simply reproducing the seasonal cycle. The following discussion will focus on the predictability of SST, bottom temperature, and bottom oxygen concentrations at all three locations discussed previously (Figs 7, 8, 9), as well as within the entire model domain (Fig. 10), over the upwelling season (April–September), specifically the skill of the forecasted anomalies from monthly climatology. Note that the January initialized forecasts have a longer lead time than the April initialized forecasts.

While most of the SST anomalies are poorly forecasted by J-SCOPE, the re-forecasted anomalies for SST during 2009 were well correlated with hindcast anomalies (R > 0.5) on the shelf throughout the upwelling season (May–September). SST was systematically too high in all the forecasts (Figs 7 and 8 Table 1), which was probably due to the bias in the shortwave radiation known to exist in this large-scale climate model (Fig. 3). The model performed the worst in the April 2014 forecast. A map of the modeled performance comparing the forecasted anomalies to the hindcasted anomalies over the upwelling season, plotted as R, confirms these patterns of skill throughout the domain (Fig. 10). The regional model forecast was able to reproduce the variability, and in most forecasts, perform on par with CFS in the region (Table 1).

Consistent with the hindcast simulations, forecast model performance was better for bottom conditions than for SST. Forecasted bottom temperatures were biased cold in most forecasts by 0.4–2.4 degrees C (Table 1, Figure S4). Despite this bias, the forecasted bottom temperature anomalies were well correlated with the hindcast anomalies (R > 0.5) on the outer shelf during the upwelling season (Figs 7 and 8, Table 1), except for the April 2014 forecast. Some of the forecasts had R values better than our significance threshold (R > 0.5), but had non-significant RMSD values indicating that the forecasts had the correct phasing but with a different amplitudes than the hindcasts (Table 1). Bottom temperature forecasted anomalies perform better on the mid-shelf (CE042) than at the shallower site (CE015, Table 1). This pattern emerges in the spatial maps of R between the forecasted and hindcasted anomalies of bottom temperature, as well (Fig. 10).

On the Oregon shelf, the temperature profile comparisons along the Newport Line were forecast skillfully (RMSD < 1, R > 0.5) for half of the realizations (Fig. 9, Table 1). The vertical structure of the water column is captured by the forecast when averaged over the entire upwelling season (Table 1), in addition to seasonal anomalies (Fig. 9). As discussed, the fall transition is difficult to forecast on these timescales, so the lack of skill in the forecast during this time is not surprising.

The model demonstrated the most predictability for oxygen, both in terms of phasing (R) and overall variability (RMSD). Bottom oxygen forecasted anomalies were reasonably well simulated for all the locations (Table 1). Similar to the other ocean conditions, R > 0.5 suggest that phasing of the forecasts is correct, even if RMSD > 1 suggests that the variability (e.g. amplitudes) differ (Table 1) for some of them. One of the forecasts had significant RMSD values, but non-significant or negative R-values indicating that the forecasts had the right variability but was not in phase with the hindcasts. In general, the forecasts were biased low. This bias was most likely due to strong, persistent forecasted upwelling-favorable winds, as relaxations have been shown to be important to relieve hypoxia^43^,^44^ and the forecasted winds from this year did not experience the observed frequency of relaxations (Fig. 3). Overall, the forecasted bottom oxygen anomalies were skillfully represented throughout the model domain (Fig. 10). While all the forecasts performed the worst in 2014, and in the shallowest region of the shelf, they still maintained broad areas of skillful forecasts (Fig. 10, Table 1).

On the Oregon shelf, the forecasts of vertical profiles of oxygen at the mid-shelf location of the Newport line (NH10) resemble their observed counterparts in terms of averages over the entire upwelling season (Table 1). The model performs the worst in the spring and fall months (Fig. 9). The fall transition is difficult to predict on seasonal timescales.

**Figure 11:**
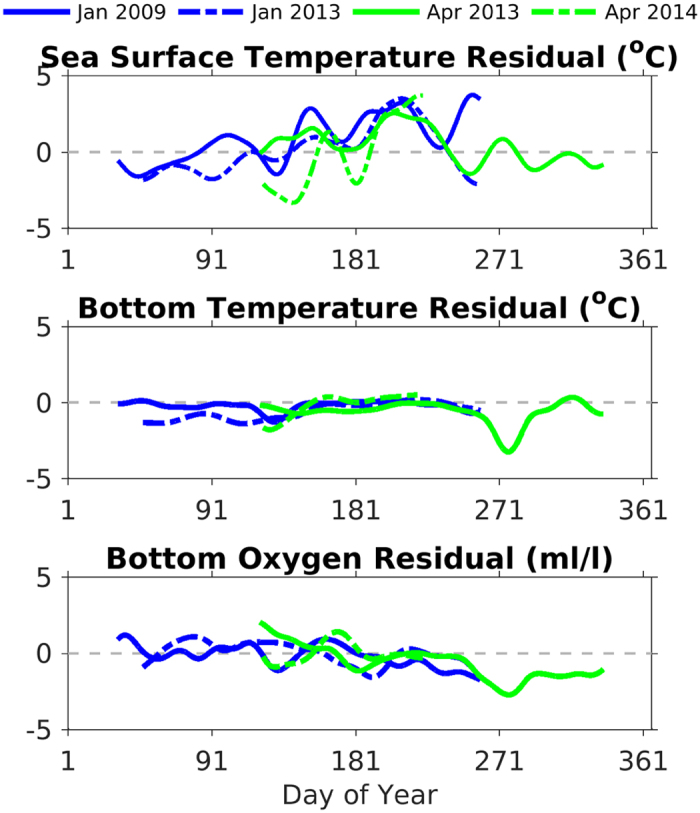
Time series of the residuals of the forecasted and hindcasted anomalies (i.e. forecast anomaly – hindcast anomaly) from the OCNMS mooring location, CE042, off the Washington shelf in 42 m of water. The x-axis is day of year. The anomalies use the 2009–2014 hindcasts averaged together as a reference climatological field shown in Fig. 5. All four forecasted anomalies from Table 1 are plotted here. January- initialized forecasts from 2009 (solid) and 2013 (dashed) are blue. April-initialized forecast from 2013 (solid) and 2014 (dashed) are green. The time series have been smoothed with a 30-day filter (a) SST, (b) bottom temperature, and (c) bottom oxygen (ml/l).

When the residuals of the forecasted anomalies from the hindcast anomalies are plotted in time, for all four forecasts shown in Table 1, the predictability of ocean conditions over seasonal timescales becomes more evident. Both the January- and April-initialized forecasts have minimal residuals (<1) for bottom temperature and bottom oxygen until September (Day of Year 270, Fig. 11). For SST, the residuals vary more over the time series at this mid-shelf location (CE042).

Overall, J-SCOPE does have skill in forecasting beyond the seasonal cycle, but the predictive skill of J-SCOPE depends on the parameter of interest, the time of year, and the location in the domain. The results from these four forecasts are promising and indicate that seasonal forecasts in this region of these ocean conditions for fisheries are possible.

### Aragonite saturation forecasts

Empirical relationships^32^ using temperature and oxygen to estimate aragonite saturation state were employed to create aragonite saturation maps and cross-sections from the April forecasts of the August cruise period from 2013. Forecasts are compared to aragonite saturation observations from the cruise (Figure S5).

**Figure 12:**
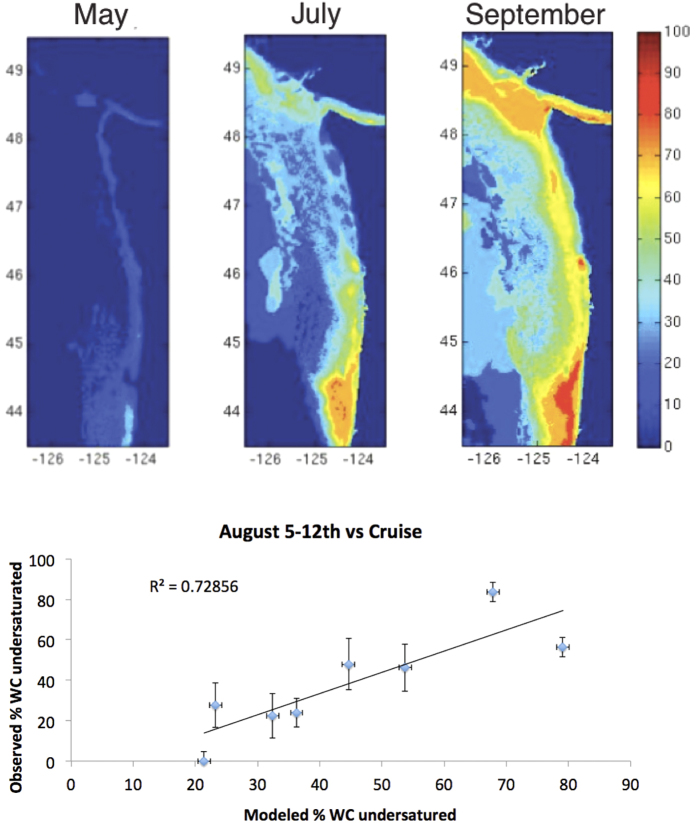
Maps of the percent of upper 100 m of water column that is undersaturated with respect to aragonite for the April-initialized forecast of May –August conditions (top). The lower panel compares the observed percentage in August to the forecasted percentage. Figure generated using Matlab version 2015b (http://www.mathworks.com/products/new_products/latest_features.html) and Adobe Illustrator CS5 (http://www.adobe.com/products/illustrator.html).

Over the upwelling season, the smallest amount of undersaturated water is present at the onset of upwelling, and it evolves over the ensuing months until it reaches a maximum prior to the fall transition. Despite small biases discussed in the SI, the forecast is able to simulate the spatial gradients of the increasing percentage of the upper water column that is undersaturated when compared with observations (Fig. 12). The shelf water feeds the estuaries, which feature shellfish aquaculture throughout the upwelling season. Regionally, Heceta Bank (44–45° N) in Oregon and the region corresponding to the Juan de Fuca Eddy, just outside the mouth of the Strait of Juan de Fuca, both experience the highest percentage of undersaturated water in the upper 100 m of the water column. These regions correspond to hot spots for respiration associated with retentive zones^31^. In addition, they correspond to regions that experience the most intense dissolution of pteropods (calcifying zooplankton) collected during a cruise in 2011^34^.

## Discussion

The J-SCOPE forecast system relies upon the following components: a real-time observational network, a working hindcast simulation of the seasonal patterns for the region complete with biogeochemistry, a region with significant skill from a global seasonal forecast system like CFS, and an identified group of stakeholders with products designed in mind for them. For J-SCOPE, NANOOS provides a portal for real-time regional observations. Regional hydrodynamic hindcast models have been developed to understand the dynamics on the shelf with success, such that biogeochemical models can be designed and linked to them as well^24^,^30^,^31^,^45^,^46^,^47^. NANOOS and the California Current Integrated Ecosystem Assessment^3^ bring linkage to and feedback from resource managers and other stakeholders with interest in these oceanographic and biogeochemical models.

J-SCOPE results indicate the forecasts have predictive skill on the order of several months into the future from late winter conditions through much of the upwelling season. The best skill lies on the outer shelf and near the bottom, mostly because of the biases at the surface, specifically due to excess solar radiation and wind in CFS on these timescales. Sensitivity tests to the shortwave forcing performed on the hindcast simulations revealed that a 20% reduction in the shortwave forcing reduced the SST by 2–3 degrees C on average, which is enough to account for the bias in 2013. The strengths of the model include the bottom temperature and bottom oxygen forecasts on the mid to outer shelf, and the spatial variability of those conditions over the upwelling season. Certain dynamics are less predictable on these timescales, and these limitations should be kept in mind. Specifically, the fall transition, which brings the end of the upwelling season, appears to be poorly predicted. This recurring problem means that the cumulative upwelling index will be too high, duration of hypoxic or ocean acidification events will be too long, and the severity of events will be exaggerated. This problem could be rectified through a combination of forecasts with increasing time resolution combined with an observations network. The resulting toolbox could prove useful for management decisions. A complete characterization of forecast skill would require a more extensive forecast suite and longer time series consisting of moored observations that span the entire year.

As an example of the potential utility for J-SCOPE, a recent study^22^ used it to forecast the spatial distribution of Pacific sardine, whose migration and spawning area respond directly to surface temperature and other ocean conditions^48^,^49^. The study fit generalized additive models relating 2009 ocean conditions (SST, salinity, and chlorophyll), as reforecast by J-SCOPE, to observed spatial distributions of sardines. Notably, the authors^22^ found that using the 2009 reforecasts initialized in January had moderate ability to predict May–August sardine distributions from three surveys. Their method accounts for the warm bias in surface waters by fitting sardine presence to J-SCOPE reforecasts before using the forecasts to predict future sardine distribution. Ongoing research aims to extend the forecasting to other species known to respond to ocean conditions such as tunas^50^, Pacific hake *Merluccius productus*^51^, and salmon *Oncorhynchus* spp.^52^,^11^.

J-SCOPE development is ongoing, and some technical improvements target some of the limitations identified above. Specifically, CFS contains a combination of biases in the wind stress and cloud fields leading to over-estimation of insolation^53^. Future efforts might consider dynamical downscaling with a high-resolution coupled atmosphere-ocean model^53^,^54^ in order reduce the biases outlined here. We will continue to test the forecasts in order better understand our capabilities and to convey uncertainties in the forecasts. In addition, we are including ensembles of multiple model runs in our future forecasts to better gauge uncertainty.

Having begun experimenting with seasonal forecast capabilities, we see strong potential to translate ongoing J-SCOPE forecasts into products relevant to US West Coast commercial fisheries, focusing on the phenomena that trigger stakeholder decisions and for which J-SCOPE forecasts have the most skill. In particular, we see potential utility for fisheries targeting pelagic species that respond strongly to temperature (e.g. hake, tuna, and mackerel in addition to sardine), since fishermen typically follow water temperatures when making decisions about fishing location^55^ and in at least one case are already using short-term (48 hr) forecasts (http://nvs.nanoos.org/TunaFish).

Our results suggest that J-SCOPE has skill to predict the location and onset of hypoxia. The onset of hypoxia is sufficient information for state and tribal crab managers to prohibit or warn against the setting of traps similar to closures already triggered by harmful algal blooms (http://wdfw.wa.gov/news/aug0415a/). Additionally, the availability of empirical relationships for aragonite saturation^32^ allowed us to relate ocean conditions to aragonite saturation state that will provide useful predictions for shellfish operations. Specifically, our results suggest that J-SCOPE has skill to predict corrosive bottom-waters on the shelf just outside estuaries with shellfish operations. Shellfish aquaculture is particularly sensitive to corrosive conditions.

Further work with commercial fisheries and fishery managers will require direct engagement with stakeholders to understand these critical decisions and time scales for these decisions^21^. We expect seasonal forecast systems to have broad applicability to stakeholders and managers in other regions, similar to existing forecast systems for algal blooms and pathogens^56^,^57^.

## Conclusions

Experiments with J-SCOPE suggest seasonal forecasting of regional ocean conditions for fisheries and managers is possible with the right combination of components. Those components include regional predictability on the seasonal timescale from a large-scale model of the physical environment (e.g. SST, winds), a high-resolution regional model with biogeochemistry that simulates regional seasonal conditions in hindcasts, a working relationship with local stakeholders, and a real-time observational network. Through the experiments described here, we discovered that biases in the shortwave radiation and the winds from the CFS model present challenges to forecasting on seasonal timescales. Despite these challenges, results suggest J-SCOPE forecasts have skill on timescales up to four months depending on the variable and time of year. We are working through NANOOS to socialize such results with regional managers and fishers to gain their feedback on the utility of such information.

One objective of this paper is to encourage other research teams to make seasonal forecasts of direct relevance to marine ecosystems, by coupling CFS (or other seasonal forecasts) to the many existing regional models of physical oceanography (e.g. ROMS, HYCOM, FVCOM), partnering with a real-time observational network, and seeking out local stakeholders and fishers of interest. The vision for the Global Ocean Acidification Observing Network (GOA-ON) highlights the mutually beneficial relationships among real-time observing, forecasting, and stakeholder communities^58^,^59^. Regional models that include plankton dynamics and biogeochemistry facilitate predictions beyond physics, reaching additional users and stakeholder needs. Multiple efforts and approaches, and in particular the lessons learned from different systems and regions, will be the best way to make progress in this arena.

## Additional Information

**How to cite this article**: Siedlecki, S. A. *et al*. Experiments with Seasonal Forecasts of ocean conditions for the Northern region of the California Current upwelling system. *Sci. Rep*. **6**, 27203; doi: 10.1038/srep27203 (2016).

## Supplementary Material

Supplementary Information

## Figures and Tables

**Table 1:**
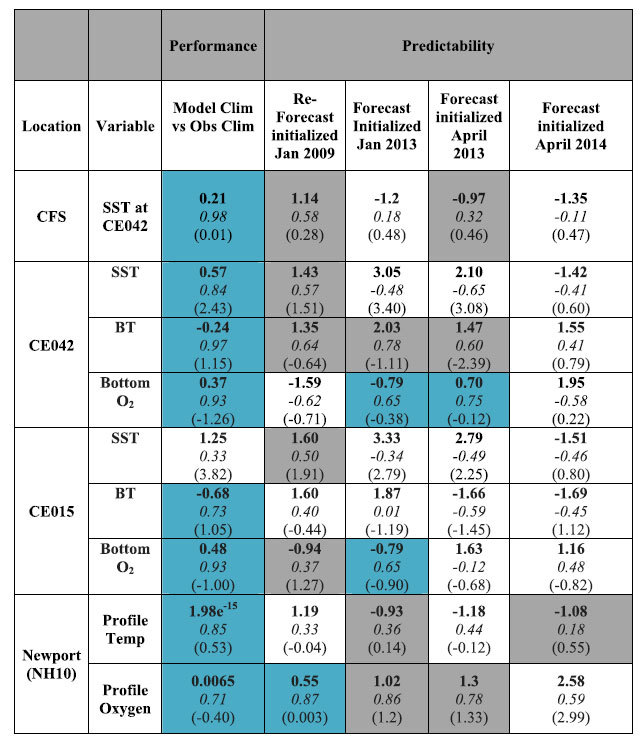
Summary of statistics for “Performance” and “Predictability” for the forecast and hindcast simulations of the J-SCOPE forecast system averaged over the upwelling season (April–September).

The RMSD is indicated in bold, the R-value is indicated in italics, and the bias is in parentheses. The results are shown for four locations: the Washington shelf as represented by the coarse-scale CFS model (CFS), Washington mid shelf (CE042), Washington inner shelf (CE015), and Oregon outer shelf (NH10). The column *‘Model Clim vs Obs Clim’* is a metric of “Performance”, comparing the hindcast simulated climatology to the observed climatology on the same timeframe as the samples (see Sup. Table 1). Other columns are measures of “Predictability” comparing forecast anomalies to the hindcast (or reanalysis for CFS) anomaly. For the profiles, the anomalies were averaged over depth to get one value for each observation, and then correlations were made based on these depth-averaged anomalies. Each row displays a different model field (SST, bottom temperature (°C) or bottom oxygen (ml/l)). Teal shaded boxes indicate performance or predictability better than our skill thresholds (−1<RMSD<1 and R > 0.5). Grey shaded boxes indicate that either R or RMSD are better than the skill threshold, but not both.
