# Supplementary material for: Experiments with Seasonal Forecasts of ocean conditions for the Northern region of the California Current upwelling system
Source: Sci Rep. 2016 Jun 7;6:27203. doi: 10.1038/srep27203 (PMC4895184; doi:10.1038/srep27203)
Supplement: Supplementary Information [file srep27203-s1.pdf]

Experiments with Seasonal Forecasts of ocean conditions for the Northern region of the California Current upwelling system

SAMANTHA A. SIEDLECKI (siedlesa@uw.edu**)[1*], ISAAC C . KAPLAN (**isaac.kaplan@noaa.gov**)[2], ALBERT J. HERMANN (**albert.j.hermann@noaa.gov**)[1,6], THANH TAM NGUYEN (**nttam15@uw.edu**)[1], NICHOLAS A. BOND (**nicholas.bond@noaa.gov**)[1,6], JAN A. NEWTON (**janewton@uw.edu**)[3], GREG D. WILLIAMS (**greg.williams@noaa.gov**)[4], WILLIAM T. PETERSON (**bill.peterson@noaa.gov**)[5], SIMONE R. ALIN (**simone.r.alin@noaa.gov**)[6], RICHARD A. FEELY (**richard.a.feely@noaa.gov**)[6]**

1. *Joint Institute for the Study of the Atmosphere and Ocean, University of Washington, Box 355672, 3737 Brooklyn Ave NE, Seattle WA 98195 USA*
2. *Conservation Biology Division, Northwest Fisheries Science Center, National Marine Fisheries Service, National Oceanic and Atmospheric Administration (NOAA), 2725 Montlake Blvd E, Seattle WA 98112 USA*
3. *Applied Physics Laboratory, University of Washington, 1013 NE 40th St, Box 355640, Seattle, WA 98105 USA*
4. *Pacific States Marine Fisheries Commission, under contract to Northwest Fisheries Science Center, National Marine Fisheries Service, NOAA, 2725 Montlake Blvd E, Seattle WA 98112 USA*
5. *Fish Ecology Division, Northwest Fisheries Science Center, National Marine Fisheries Service, NOAA, 2725 Montlake Blvd E, Seattle WA 98112 USA*
6. *NOAA, Pacific Marine Environmental Laboratory, NOAA, 7600 Sand Point Way NE, Seattle WA 98115 USA*

*siedlesa@uw.edu *(206)616-7328, Box 355672, 3737 Brooklyn Ave NE, Seattle WA 98195 USA*

Supplemental Information:

Model Climatology:

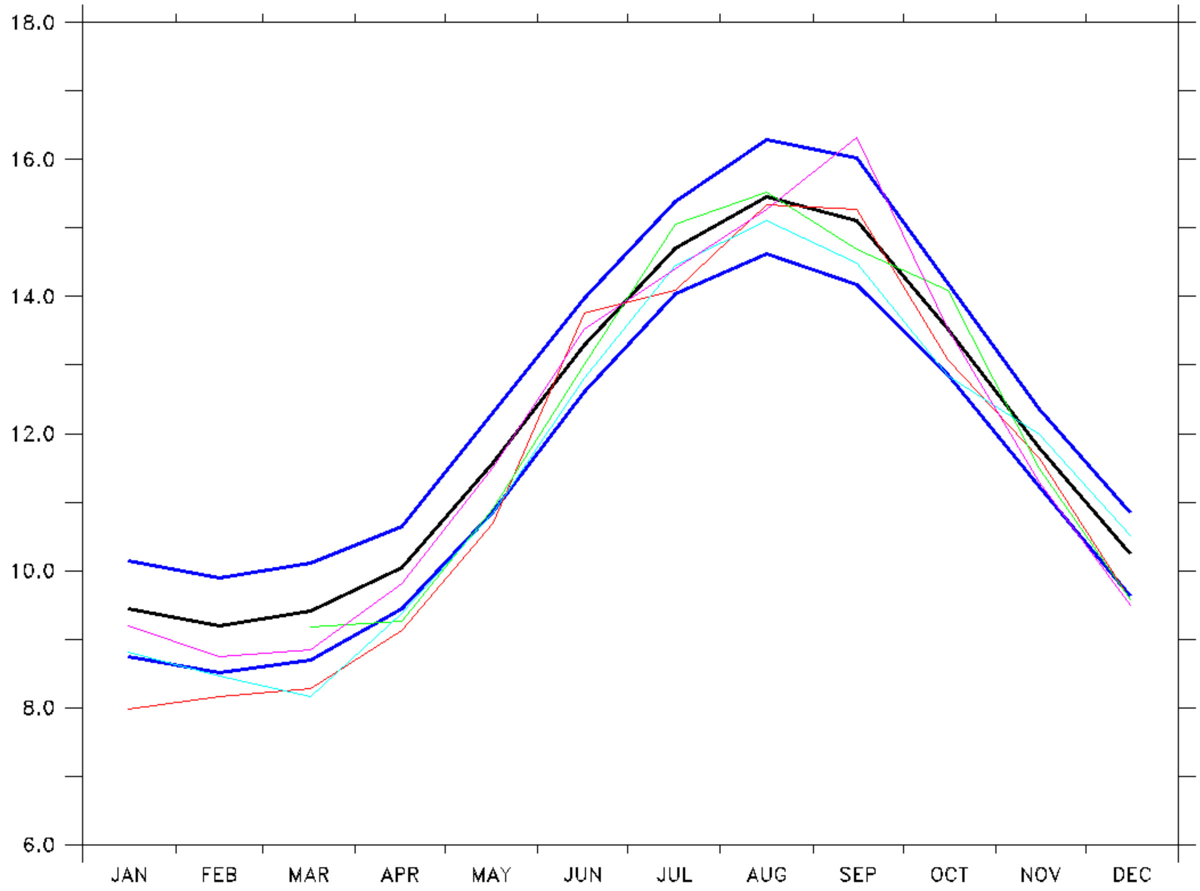

Figure S1: Monthly average SST (degrees C) from CFS analysis, spatially averaged over the J-SCOPE domain. We compare SST values for 2009 (thin red line), 2011 (thin green line), 2012 (thin light blue line), and 2013 (thin purple line) with a monthly climatology calculated from CFS reanalysis of years 1979–2009 (thick black line). Thick blue lines indicate +/- 1 std of the monthly values for 1979–2009.

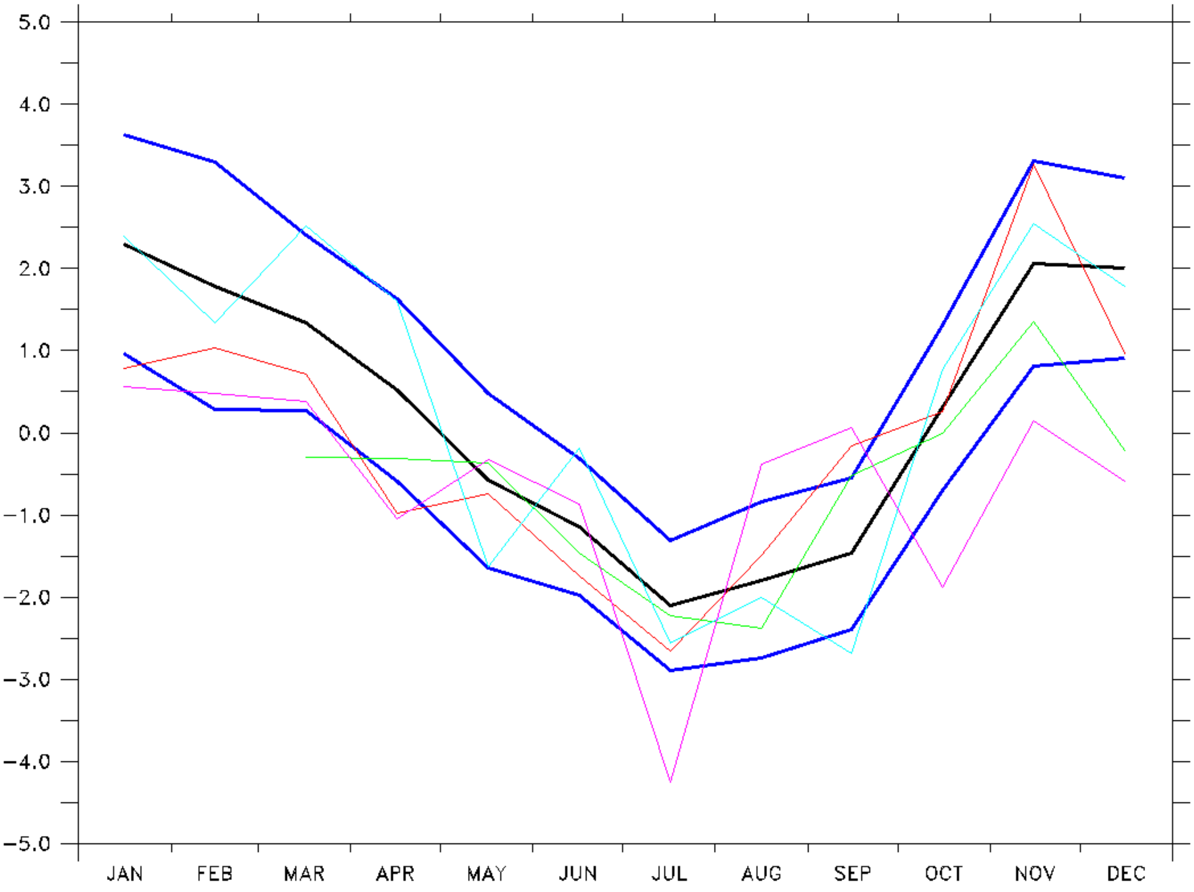

Figure S2: As in Figure S1, for alongshore (North/South) monthly average wind speed (m/s), spatially averaged over the J-SCOPE domain.

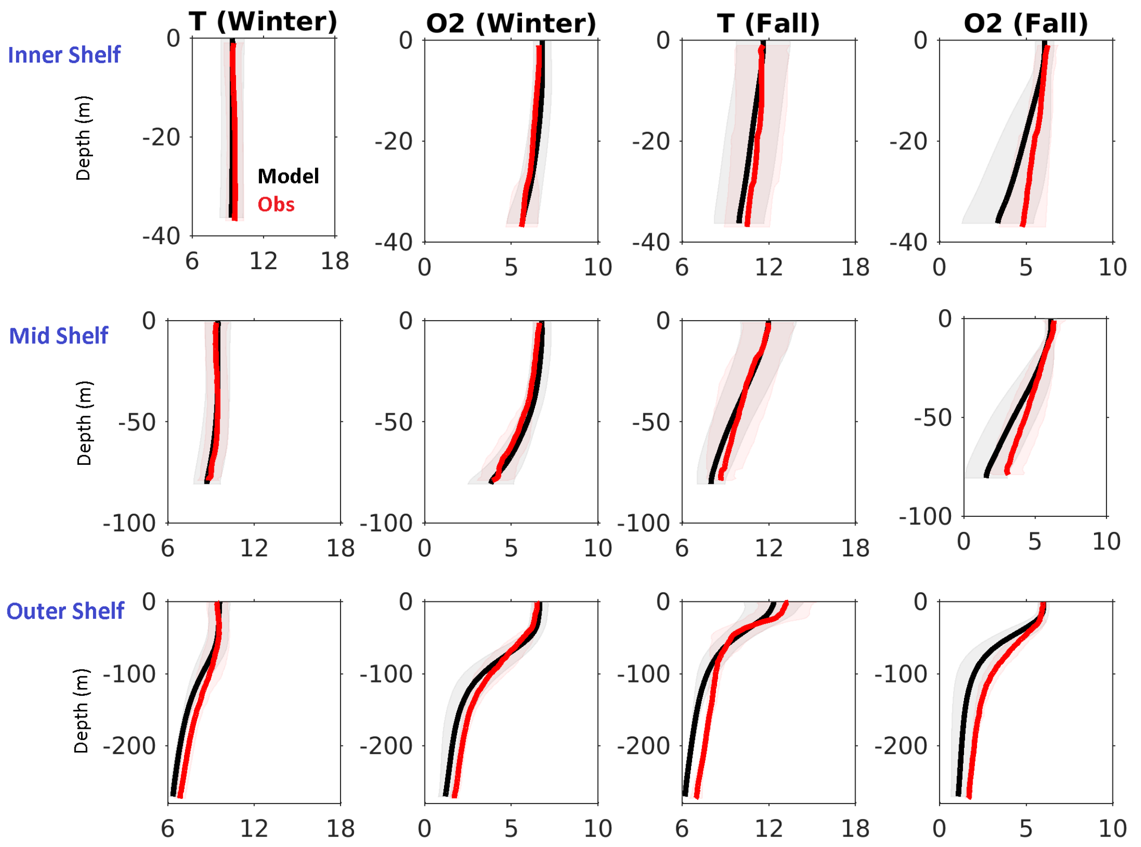

Figure S3: A comparison of model and observational climatologies from three locations (inner, mid, and outer shelf) along the Newport Line on the Oregon shelf. The model climatology (black) is the average of the 2009–2014 hindcasts, at each location and depth. The observational climatology (red) is based on twice-monthly samples. The standard deviation around each climatology is shaded in the background. Temperature and oxygen profiles are shown from winter (January-March) and fall (October-December). Statistics summarized in Table 1.

Model Skill Statistics:

Three skill metrics were used to evaluate the performance and predictability of the simulations presented here. The equations for each of the metrics are after Jolliff et al. 2009, and detailed below.

$$RMSD^* = \left[\left(\frac{1}{N}\sum_{n=1}^{N}[(m_n - \bar{m}) - (r_n - \bar{r})]^2\right)^{0.5}\right] * sign(\sigma_m - \sigma_r)(\text{Eq. } 1)$$

$$RMSD = \frac{RMSD^*}{(\sigma_r)} \text{ (Eq. 2)}$$

$$Bias^* = \bar{m} - \bar{r} \text{ (Eq. 3)}$$
$$Bias = \frac{Bias^*}{(\sigma_r)} \text{ (Eq. 4)}$$
$$R = \frac{cov(m,r)}{(\sigma_m \sigma_r)} \text{ (Eq. 5)}$$

where m indicates the model field, r indicates the reference field, an overbar indicates the average, σ is the standard deviation.

Model Performance:

Below we illustrate some preliminary tests of model performance, without removing the seasonal cycle (i.e. without calculating anomalies). In the main text we present more rigorous tests after removing the seasonal cycle.

Key variables from the model were the focus of this analysis: SST, bottom temperature (BT), and bottom oxygen. The information is synthesized in Table 1.

In 2013, the model accurately forecast the location and timing of the onset of hypoxia. At the Cha'ba buoy (~48 N in 90 m of water), on the northern end of the Washington shelf, the forecast predicted that hypoxia would not develop over the 2013 upwelling season (Figure S3). Except for two short events (about 5–6 days each) the location did not go hypoxic. At the Cape Elizabeth OCNMS mooring in 42 m of water (~47 N), the forecast predicted the onset of hypoxic conditions (<1.4 mL/ L oxygen) on 27th of June (April forecast), or the 4th of July (Feb forecast). This location observed hypoxia on 11th of July. The forecast predicted the location would remain hypoxic and develop anoxia in September. The observations show that while this location remained hypoxic until the 5th of September, the location never went anoxic.

Aragonite Saturation State Forecasts:

Two depth intervals are qualitatively compared to the observations, the surface as well as 60 decibars (db) (approximately 60 meters) below the surface. The model captures the latitudinal trend in the region- more undersaturated water is present on the Oregon shelf. One bias is that the model underestimates the saturation state of waters at 60 db. This is the result of the model's intense upwelling favorable winds with few relaxations, or reversals. Relaxations in the winds would prevent anoxia from developing[43, 44], and without these relaxations, the model is biased low in oxygen and thus produces water more corrosive than observed. The second bias is that the model misses the influence of the Columbia River plume in the region, especially at the surface. The Columbia River plume is known to be corrosive[60] due the outflow of low pH, low alkalinity freshwater, but the model misses the presence of this low pH water. This is because the empirical relationship predicting saturation state is not calibrated for low salinities (<20 psu), and because the riverflow comes from a climatology.

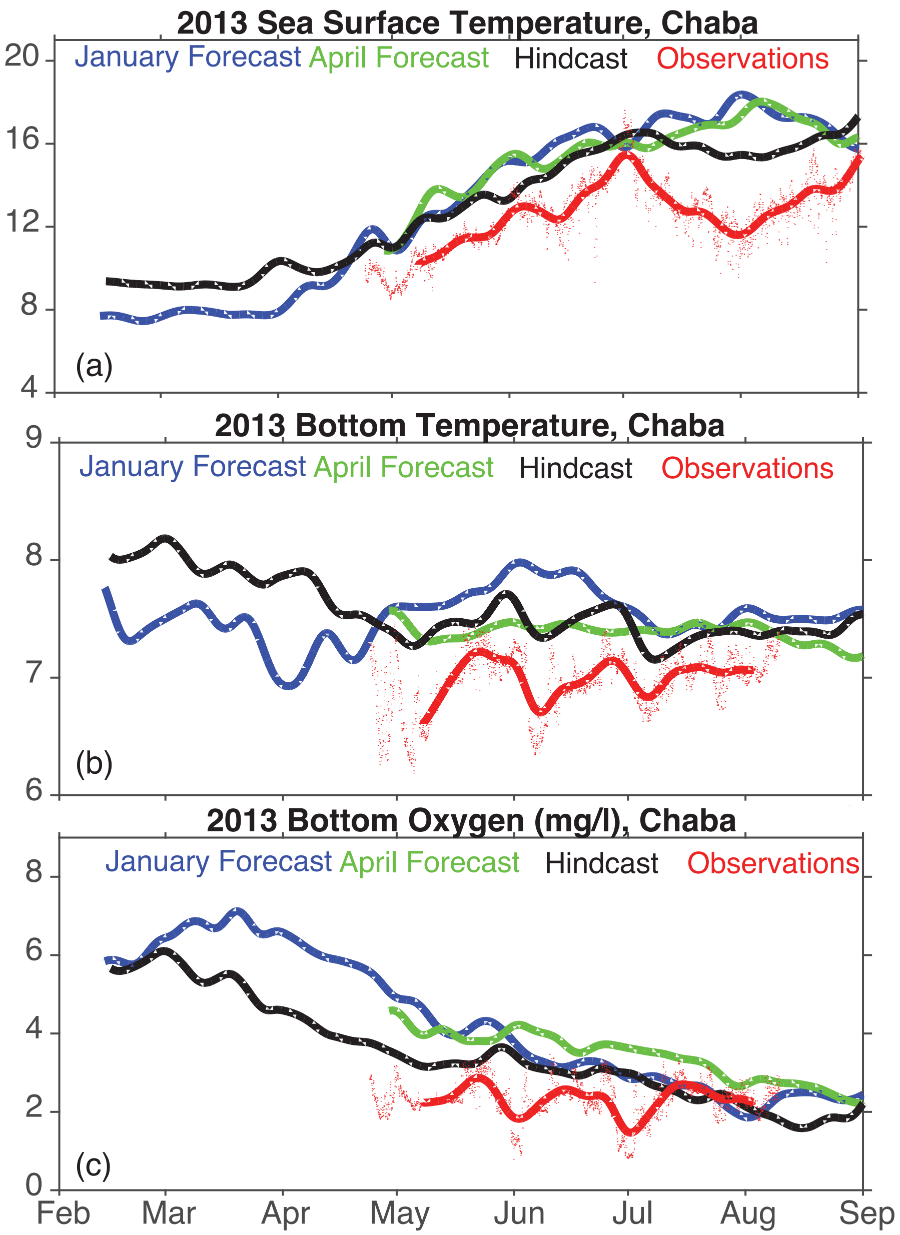

Figure S4: Model comparisons to observations (red) made at the Cha'ba buoy in 2013. The model was run in both hindcast (black) and forecast mode (green, blue). The data were filtered with a running filter over 14 days for both the model and the observations. All three panels are from a site off the coast of Washington at La Push (48 deg N) in 90 m of water (a) SST (b) bottom temperature, (c) bottom oxygen.

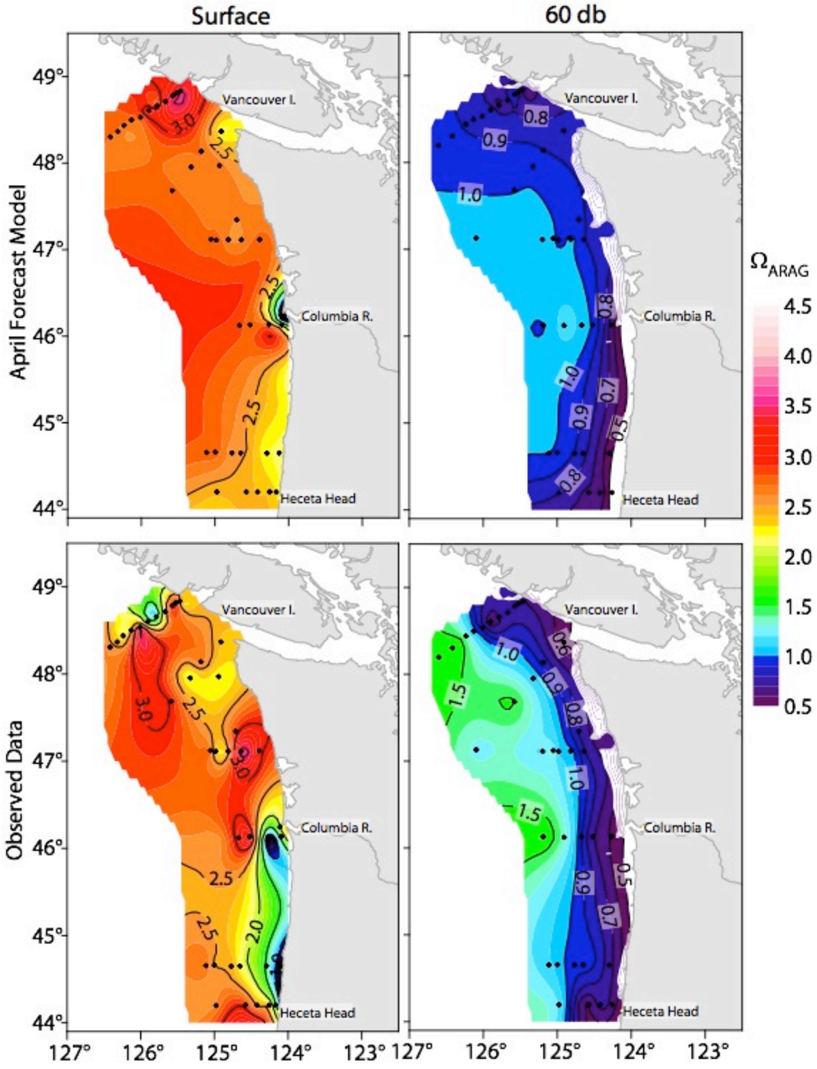

Figure S5: Maps of aragonite saturation state at the surface (left) and 60 db (right) for the April forecast of August conditions (top) and the observed conditions in August, 2013 (bottom). 60db is approximately 60m. Figure generated using Surfer version 13 (http://www.goldensoftware.com/products/surfer).

	Source	Temperature		Oxygen	
**Location**		**Used data**	**Frequency**	**Used data**	**Frequency**
**CE015**	OCNMS	202,194	10 minutes	162,498	10 minutes
**CE042**	OCNMS	214,157	10 minutes	183,302	10 minutes
		**Profiles (Number of Data Points)**	**Frequency**	**Profiles (Number of Data Points)**	**Frequency**
**NH03**	Newport Line	130 (5,829)	0 -4 profiles a month	128 (5,692)	0 - 4 profiles a month
**NH10**	Newport Line	114 (9,096)	0 - 3 profiles a month	113 (8,937)	0 - 3 profiles a month
**NH25**	Newport Line	98 (27,529)	0 - 5 profiles a month	96 (26,982)	0 - 5 profiles a month
		**SST**		**Bottom Temperature (70 m)**	
**Location**		**Used data**	**Frequency**	**Used data**	**Frequency**
**NH10**	Newport Line	18,834	1 hour	18,258	1 hour

Supplementary Material Table 1: Summary of observations used to validate the model over the upwelling season (April–September). The observations are shown for the following locations: Washington mid shelf (CE042), Washington inner shelf (CE015), and Oregon shelf – inner (NH05), mid (NH10) and outer (NH25).
